# Endovascular and Endoscopic Treatment of Hemobilia: A Report of Two Cases

**DOI:** 10.7759/cureus.28383

**Published:** 2022-08-25

**Authors:** José D Cardona, Oscar M Rivero, Renzo Pinto, Camilo A Barragán, David F Torres

**Affiliations:** 1 Radiology, University Hospital Fundación Santa Fé de Bogotá, Bogotá, COL; 2 Gastroenterology, University Hospital Fundación Santa Fé de Bogotá, Bogotá, COL

**Keywords:** computed tomography (ct ), endovascular procedure, upper endoscopy, hemobilia, gastrointestinal bleeding

## Abstract

Hemobilia is a term used to describe bleeding caused by abnormal communication between blood vessels and bile ducts. Some vascular anomalies, such as aneurysms or arterio-biliary fistulas, facilitate the appearance of this type of biliary bleeding. Other causes have been described such as iatrogenic causes secondary to percutaneous procedures, infections, tumors, and trauma. We report two cases of hemobilia. The first one presented with acute biliary bleeding with secondary hypovolemic shock. Bleeding was controlled after percutaneous interventions with a selective embolization technique. The second case was a patient who presented to the emergency department after a fall from his height. During hospitalization, acute cholangitis was documented, associated with hemobilia. A wide papillotomy and biliary duct instrumentation were done with the extraction of a large blood clot. Angiography is the standard for diagnosis and embolization becomes the best tool for the detection and control of vascular abnormalities that can perpetuate bleeding.

## Introduction

Bleeding originating from the bile duct (hemobilia) has been described since the middle ages in cadaver revisions of patients with digestive bleeding [1]. At the moment, with the uprising of new technologies and a better understanding of the pathophysiology of biliary pathology, new causes of this rare problem have been described as well as its relationship with some percutaneous and endoscopic biliary procedures [2]. Some vascular anomalies facilitate the appearance of this type of biliary bleeding such as aneurysms or arterio-biliary fistulas [3]. Given their arterial origin, these hemorrhages can be life-threatening and may condition the need for immediate therapeutic action [4]. Other causes of hemobilia have been described such as iatrogenic causes secondary to percutaneous procedures, infections, tumors, and trauma [5].

We report two cases of hemobilia. The first one presented with acute biliary bleeding of arterial origin with secondary hypovolemic shock. Bleeding was controlled after percutaneous interventions with selective embolization technique as described in other publications [6]. The second case was a patient who presented to the emergency department after a fall from a height. During hospitalization, acute cholangitis was documented according to the 2018 Tokyo Guidelines, associated with hemobilia [7]. 

## Case presentation

Case 1

A 58-year-old female patient presented with acute abdominal pain of high intensity, located in the epigastrium, radiating to the lumbar region. It was associated with nausea; however, no other symptoms were documented. Paraclinical studies did not show leukocytosis or anemia. Liver enzymes showed normal bilirubin with elevated transaminases and alkaline phosphatase. Hepatobiliary ultrasound evidenced biliary sludge in the common bile duct (CBD). 

The patient had a history of cholecystocholedocholithiasis in February 2020. An endoscopic retrograde cholangiopancreatography (ERCP) was performed followed by papillotomy and pancreatic stent insertion as a prophylactic procedure. A 5 mm calculus and biliary sludge were removed from the CBD. A plastic stent 10 Fr x 7 cm was placed for acute cholangitis drainage. The procedure was performed without complications. Subsequently, the patient was taken for a laparoscopic cholecystectomy finding a pyocholecyst, omentum adhesions, and severe inflammatory changes of the structures in the Calot triangle. A Jackson-Pratt drain is left in the hepatic surgical bed and the patient went through intrahospital antibiotic treatment for five days. During her hospital stay, no complications were documented. She was discharged from the hospital with an order for biliary stent retrieval and the procedure was performed ambulatory in April 2020 using ERCP with no difficulties.

**Figure 1:**
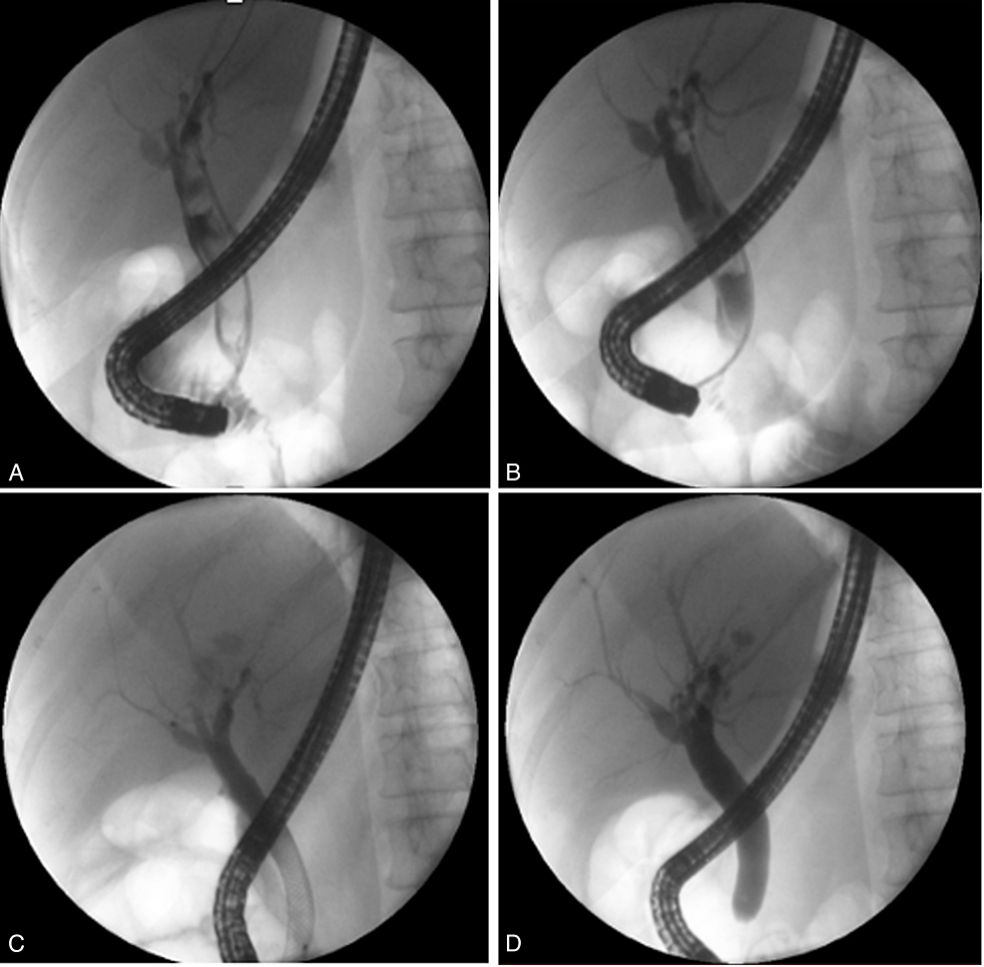
(A and B) Fluoroscopy in retrograde endoscopic cholangiography: dilatated common bile duct with blood clot mold in its interior; (C and D) Adequate drainage with full opacification in control images

**Figure 2:**
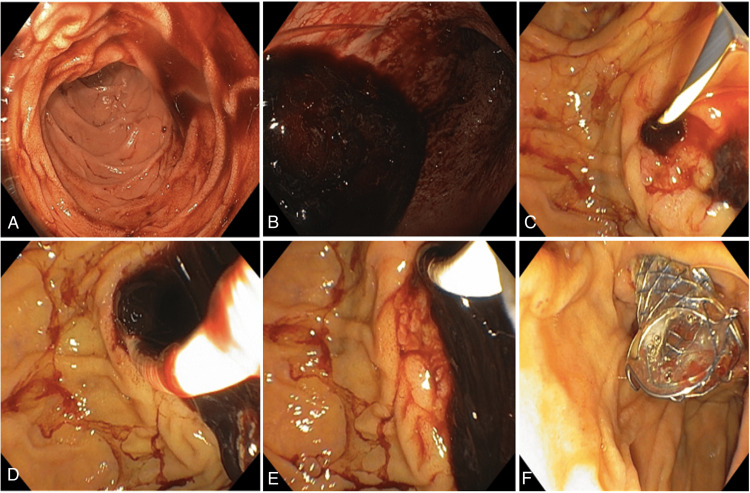
(A-E) Papillotomy and extraction of blood clot in biliary duct with posterior (F) metallic auto-expandable stent implantation with hemorrhage control

**Figure 3:**
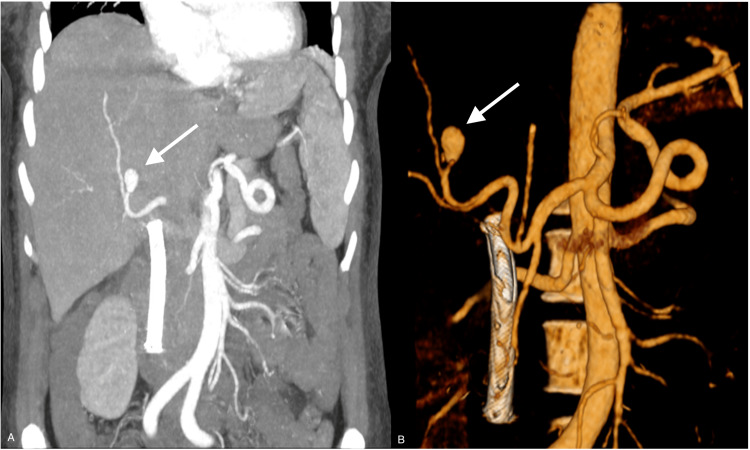
CTA (A) and 3D angiographic study (B) reconstruction of the abdomen showing the presence of a pseudoaneurysm is documented in a segmental branch of the right hepatic artery with a diameter greater than approximately 12 mm (arrows); a biliary stent previously placed in the common bile duct can also be seen CTA: computed tomography angiography; 3D: three-dimensional

**Figure 4:**
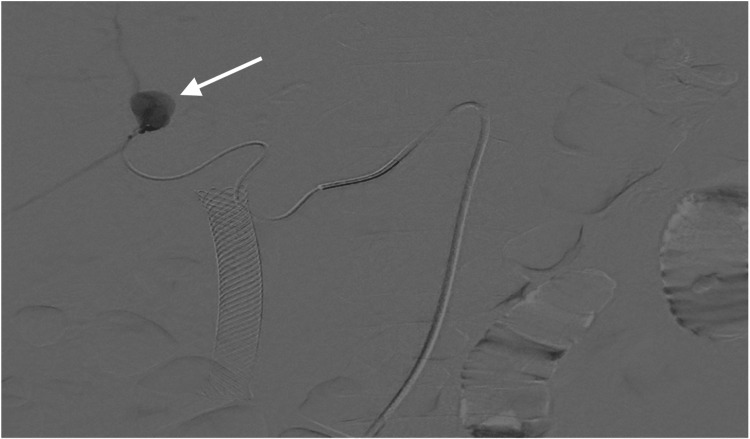
Digital subtraction angiography image showing selective injection through a microcatheter and identifying the presence of a pseudoaneurysm of a segmental branch of the right hepatic artery (arrow). Active contrast medium extravasation is not documented.

**Figure 5:**
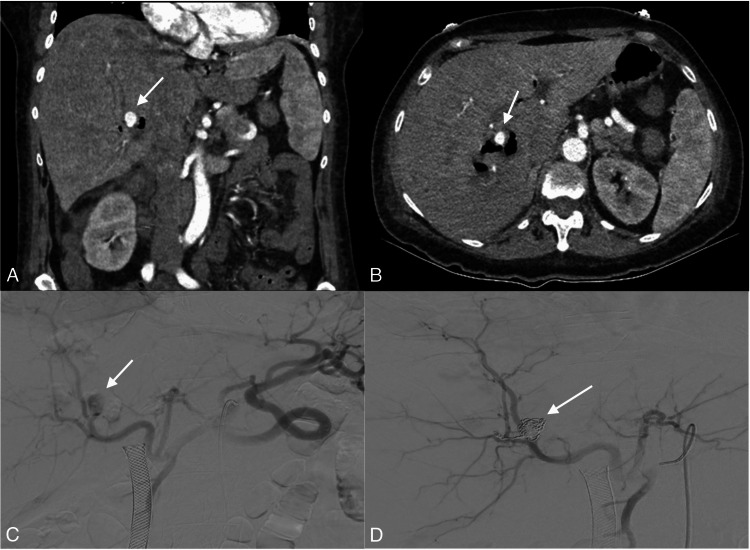
(A and B) Contrast tomography of the abdomen in coronal and axial views of the arterial phase with evidence of aneurysmal lesion (arrows) dependent on the right hepatic artery with signs of bleeding, (C) Fluoroscopy with angiographic evidence of an aneurysmal lesion of the right hepatic artery (arrow), and (D) subsequent successful embolization with coils (arrow)

The patient returned to the emergency room with symptoms consistent with residual choledocholithiasis. The ERCP that was performed on the patient's readmission showed known changes from the previous endoscopic procedure (papillotomy) and blood clots. Intrahepatic bile ducts were normal. Extrahepatic bile ducts were dilated with a 14 mm CBD with multiple filling defects (Figure 1). At instrumentation, blood clots, biliary sludge, and microcalculi were extracted (Figure 2). The cause of the hemobilia was not identified in the upper digestive tract endoscopy. An abdominal computed tomography angiography (CTA) showed an intrahepatic pseudoaneurysm from a right segmentary branch of the hepatic artery (Figures 3, 4, 5). Endovascular embolization of the right intrahepatic pseudoaneurysm was accomplished with coils (Figure 5). Percutaneous drainage of two right hepatic collections was also performed with no problems. Antibiotic therapy was given for the hepatic abscess, which was positive for *Morganella morganii*. After endovascular treatment, the patient was discharged after four days. However, her evolution was torpid, presenting multiple liver abscesses, which were treated by ultrasound-guided puncture and antibiotic management. Since the last readmission, the evolution has been favorable.

Case 2

**Figure 6:**
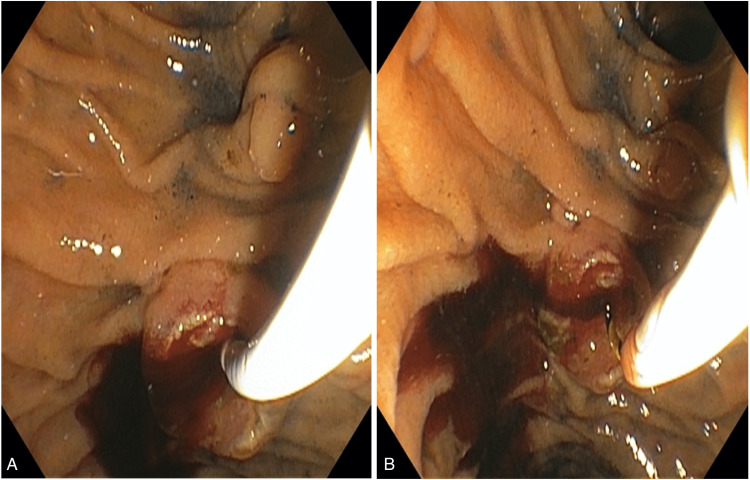
(A and B) Papillotomy and extraction of blood clot in biliary duct and bleeding control

An 84-year-old male arrived at the emergency room with a closed abdominal trauma secondary to a fall from his height with two episodes of hematemesis and abdominal pain. In admission, nonspecific CT findings were found in the gallbladder; therefore, lithiasis or neoplastic disease could not be ruled out. Complete blood count showed leukocytosis 20,300 at the expense of neutrophilia. The liver profile showed hyperbilirubinemia with a total bilirubin of 4.95, direct bilirubin of 3.64, and indirect of 1.31. Values of serum glutamic-oxaloacetic transaminase (SGOT) was 114 and serum glutamic pyruvic transaminase (SGPT) was 273. The patient presented with rapid hemodynamic deterioration with the need for vasopressor support because of persistent hypotension. The patient was transferred to the ICU. The patient was taken to an ERCP because of acute cholangitis according to the 2018 Tokyo Guidelines and for the nonspecific CT findings. The ERCP showed normal intra- and extrahepatic bile ducts (10 mm CBD). However, at the distal CBD, a filling defect was noted. A wide papillotomy and biliary duct instrumentation were done with a 9-12 mm balloon extractor with the extraction of a large blood cloth that conditioned an obstructive effect over the distal CBD (Figure 6). A lot of cloudy bile mixed with blood and microcalculi were removed. To optimize drainage, two plastic bile stents were adequately placed, one 10 Fr x 10 cm and the other 8.5 Fr x 10 cm. The patient was discharged one week after diagnostic and therapeutic ERCP. On outpatient follow-up, he recovered without recurrent bleeding or other complications.

## Discussion

Bleeding in the biliary system is called hemobilia. It is a rare cause of upper gastrointestinal bleeding. The pathophysiology occurs when there is a fistula between a blood vessel and the biliary tree. The clinical presentation may involve a triad consisting of upper quadrant abdominal pain, gastrointestinal bleeding, and jaundice. However, a small proportion of patients present all these symptoms, it depends on the underlying cause. Some cases may even present with massive bleeding, melena, or hematemesis. In addition, bleeding in the bile ducts can produce clots that obstruct these ducts, leading to elevated liver enzymes and jaundice. Previously, direct trauma (penetrating or blunt) was the most common etiology. However, today with the increasing use of endoscopic biliary procedures, iatrogenic etiology comprises about 50% of cases [8]. CTA is a diagnostic method with some advantages such as its non-invasive nature, less radiation exposure, and provision of a quick result. However, angiography is remarkably superior to CTA for detecting discreet arterio-biliary or porto-biliary fistulas and pseudoaneurysms [9]. Additionally, it is the standard for diagnosis and treatment, enabling precise characterization and localization of the bleeding source followed by immediate definitive management.

Initial management revolves around careful clinical evaluation followed by adequate volumetric resuscitation, transfusions, and general supportive care. Minor hemobilia may resolve with conservative measures. In more severe cases, once the source of bleeding is identified by imaging or endoscopy, definitive treatment should be applied. Arterial embolization is the first-line treatment in those cases of minor hemobilia that does not improve with conservatory management, or in cases of major hemobilia when transfusion is required [10]. Selective arteriography allows a precise characterization of hepatic vascular anatomy and allows the detection of a vascular lesion responsible for the acute hemorrhage. If the hemobilia is related to recent percutaneous drainage, a catheter should be moved over a wire guide before its realization, since it could hide the site of hemorrhage because of the compressive effect it can exert.

Active bleeding in angiography is seen by visualizing contrast media extravasation and its accumulation in the biliary system. Other indirect signs include the presence of a pseudoaneurysm or the detection of an arterial segment with vasospasm. It must be taken into consideration that these findings are intermittent and may not be seen during angiography. The dual vascular supply of the liver (75% portal and 25% arterial) allows for the embolization of the arterial hepatic branches with no significant ischemic deleterious effects, thus, it is important to evaluate the permeability of the portal vein previous embolization, especially in patients with liver transplant.

Once the bleeding site is identified, a supraselective embolization is recommended by releasing metallic spirals or coils through a microcatheter. The occlusion of a pseudoaneurysm requires the placement of the metallic spirals through its neck from distal to proximal. Pseudoaneurysms can keep on expanding if only their interior is filled because they do not have a true wall. Liquid embolic agents like cyanoacrylate (glue) and ethylene alcohol vinyl copolymer (Onyx®; Micro Therapeutics Inc, Irvine, California, United States) can be an option in cases with extremely tortuous arteries or when there are multiple afferent arteries involved in the pseudoaneurysm [11]. Endovascular embolization is technically effective 75-100% of the time [12]. Complications include hepatic necrosis, abscess formation, and biliary stenosis. Percutaneous embolization with thrombin injection has proven to be safe and effective and can be used alone or combined with endovascular therapy [13].

On the other hand, endoscopy has an important role in relieving obstruction caused by clotted blood products. Several endoscopic techniques can be used to achieve hemostasis of inaccessible lesions, such as dilute epinephrine application, fibrin sealant injection, clipping, thermal coagulation, balloon tamponade, and stenting. Endoscopy may precede or replace angiography in patients with hemodynamic stability without imaging evidence of intrahepatic arterial injury, or in those patients with occult upper gastrointestinal bleeding [14].

## Conclusions

Hemobilia is a rare entity that is mainly caused by aneurysms or arterio-biliary fistulas. It becomes more relevant in the context of laparoscopic or endoscopic interventions of the biliary tract. It is a life-threatening form of upper gastrointestinal bleeding that poses unique diagnostic and therapeutic challenges. Angiography is the standard for the diagnosis and treatment, as it allows for accurate characterization and localization of the source of bleeding and immediate treatment. The key to success in this pathology lies in clinical suspicion, rapid diagnosis, and timely treatment. Embolization becomes the best tool for the detection and control of vascular anomalies that can perpetuate bleeding.
